# Abstract of Proceedings of Buffalo Medical Association

**Published:** 1865-11

**Authors:** T. M. Johnson

**Affiliations:** Sec’y pro tem.


ART. II.—*Abstract of Proceedings of Buffalo Medical Association.*

TUESDAY EVENING, October 3d, 1865.

The Association met pursuant to adjournment, the President, Dr. Ring, in the chair. Present, Drs. Gay, Samo, Gould, Congar, Strong, Gleason, Little, Whitaker and Johnson.

DR. CONGAR presented the following paper:

*Defective Power of Expression in a case of Hemiplegia of the right side.*—Mrs. T. M. Y., aged forty-one years, has had eight conceptions in twenty years of married life. She has always been of a hemorrhagic temperament, which has obliged her accoucheur to give ergot before the termination of each labor, except the first two, for the purpose of securing the permanent contraction of the uterus. At the third month of the last pregnancy after a journey of leisure in a private conveyance of three or four days, on May 8th, she dined heartily; and about an hour after dinner was suddenly attacked with a most profuse uterine hemorrhage; in about thirty minutes she was completely blanched, very restless, fainting even in the horizontal position, at the wrist pulseless, and *completely hemiplegic on the right side,* although the ovum was at this time found to have been thrown off, and the hemorrhage itself to have ceased. The patient was now found to have lost nearly all ability of psychical expression. Speech, as a voluntary articulation of words for the intended expression of wants, desires, feelings, thoughts and ideas, was gone. She appeared to understand her condition, the efforts of her attendants, the language addressed to her, and spoken in her hearing, but could give no assent, no denial; could manifest uo psychical function whatever. That she wanted something, was known, by increased restlessness, turning the head on the pillow, by a gaze of the eye, by voice in the larynx, and the use of the left upper and lower extremities; but what the thing desired was, could only be learned by trying the supply of the whole circle of bodily wants upon every such effort of hers. This complete loss of the power of expression lasted, however, but about twelve hours. The use of the right lower extremity was then regained. About twenty-four hours after this, slight power over the right upper extremity appeared, and occasionally a monosyllable could be articulated; assent and denial could be perceived; and some expression of feeling could be discovered; still the face was much drawn to the left side, and the tongue when protruded turned considerably leftward. From this time, day by day, the paralysis decreased gradually, and the recovery of the power of expression steadily increased. In the progress of recuperation it was interesting to observe how much sooner the ability to *articulate* was re-established, than the power of general expression to which it is normally subservient; that is, after every word could be distinctly pronounced one after another, very few could be used at will for the manifestation of wants, feelings, thoughts and ideas.

On July 8th, two months after the attack locomotion was as perfect as ever, and the arm had nearly regained its normal use, while the hand could not pick up from the table, from a chair, or from the floor, a knife, or a fork, or a pin, or a newspaper, or a book, or even an article of wearing apparel, while it could with a pretty firm hold grasp anything put into it, which could be clapsed by the fingers and thumb. While a normal look to the right side of the face, the power of expression. and its control over articulation, on first awaking from sleep, were invariably absent; yet soon afterwards, the face became straight and had a right look, the tongue on protrusion inclined to neither side, the power of expression became nearly perfect, and its controlling influence over vocal articulation in the manifestation of psychical function became so nearly normal as at times to make one forget momentarily the previous existence of paralysis.

It is proper here to remark that we are not aware of the existence at any time of the slightest anæsthesia, of the smallest diminution or modification of any department of the sensibility of the affected side, or, indeed, of any part of the body. Three months from the date of the attack, the patient was still improving, bnt not entirely recovered; for on first awaking from sleep, when fatigued, especially by anxiety of mind, and when constipa ted, the disease still showed itself distinctly.

DR. SAMO remarked, that he attributed the abortion to the journey, and the hemiplegia to the hemorrhage.

DR. STRONG had seen cases of paralysis produced by over-eating, or by eating unwholesome or indigestable food, and thinks that might have been the cause of the paralysis in the case presented by Dr. Congar.

DR. GAY thought it might be a case of Bright's disease of the kidney.

Reports on prevailing diseases being called for typhoid fever, diarrhœa, dysentery and whooping-cough were reported as the prevailing diseases.

Miscellaneous business being in order Dr. J. J. Edmonds was proposed for membership by Dr. Congar.

There being no further business the Association adjourned.

T. M. JOHNSON, *Sec'y pro tem.*

